# Kadozan Chitosan Formulation Enhances Postharvest Quality of Fresh Indian Jujube Fruit

**DOI:** 10.3390/foods14020266

**Published:** 2025-01-15

**Authors:** Lian Chen, Yixiong Lin, Hui Li, Qingqing Liu, Yihui Chen

**Affiliations:** 1 School of Biological Science and Biotechnology, Minnan Normal University, Zhangzhou 363000, China; lian0869@163.com (L.C.); linyixiong1989@163.com (Y.L.); lihui32782752@163.com (H.L.); 2 Key Laboratory of Postharvest Biology of Subtropical Special Agricultural Products, Fujian Province University, Fuzhou 350002, China; 3 College of Food Science, Fujian Agriculture and Forestry University, Fuzhou 350002, China

**Keywords:** Indian jujube fruit, kadozan, appearance color, nutritional quality, storage shelf life

## Abstract

Indian jujube fruit is prone to perishing, resulting in a shorter shelf life after harvest. Kadozan is a liquid chitosan formulation that has a significant effect on fruit preservation. In order to explore its efficacy, the quality, and storability indicators of Indian jujube fruit were evaluated during storage at 15 ± 1 °C for 18 days. Results showed that Kadozan-treated fruit exhibited lower respiration rate, relative electrolyte leakage rate, weight loss, and decay index, along with higher firmness and commercially acceptable rate. Furthermore, Kadozan-treated fruit showed higher vitamin C, total sugar, titratable acid, total soluble solids, chlorophyll, and carotenoid contents, L* and *h*° values, but lower a* and b* values. Principal component analysis and comprehensive score revealed that Kadozan treatment helped preserve the appearance and nutritional qualities of Indian jujube fruit. The best effect was seen with 1:600 Kadozan among three concentrations (1:300, 1:600, 1:900). It was discovered that the commercially acceptable rate of 1:600 Kadozan-treated fruit was 37.5% higher than control fruit while the decay index was 30.5% lower than control fruit at 18 days. Therefore, Kadozan treatment has great substantial implications for the preservation of Indian jujube fruit, providing practical guidance for reducing its postharvest losses.

## 1. Introduction

The Indian jujube fruit is known by various names, including ber fruit, Chinee apple, desert apple, and Indian plum [1,2]. It is widely cultivated in several tropical and subtropical regions, such as China, India, Iran, Korea, Pakistan, Thailand, and Southeastern Europe [3,4,5,6,7]. Due to its crisp texture, sweet taste and high nutritional value, Indian jujube fruit is very popular among consumers [5,6]. However, the harvested Indian jujube fruit is susceptible to softening and spoilage, and its flavor and nutrition rapidly deteriorate within about a week at room temperature [3]. Severe declines occur in nutritional components like vitamin C (Vc), total sugar, and titratable acid (TA), which hampers its storage, transportation, and commercialization [3,7,8]. A variety of preservation approaches have been implemented for the preservation of Indian jujube fruit, including cold storage [9], chemical fungicide treatment [7,8,10,11], irradiation treatment [12,13], heat treatment [14], and modified atmosphere packaging [15]. Nevertheless, these preservation methods have some problems, such as being costly, having limited efficacy, and leaving chemical residues. This highlights the need for innovative, environmentally safe, convenient, and cost-efficient solutions to enhance the storability of Indian jujube fruit.

Chitosan is produced by removing some acetyl groups from chitin and is widely found in organisms like crustaceans, insects, and fungi [16,17]. Chitosan is a natural non-toxic alkaline polysaccharide with antibacterial and immune-enhancing functions [16,17]. It can be used to reduce microbial infestation and nutrient loss, thus decelerating decay rates and prolonging the storage shelf of harvested fruit [18,19,20,21]. However, chitosan has poor solubility, which may be related to its low degree of deacetylation larger relative molecular weight and high viscosity, which limits its applicability [20,21]. Therefore, it needs to be dissolved in organic acids (pH below 6.2) before use to degrade and reduce solution viscosity [22,23]. Kadozan is liquid chitosan formulation. It can be diluted with water to obtain the desired concentration without the need for acid dissolution or an alkaline solution for pH adjustment [21,22]. It exhibits environmentally friendly and biodegradable properties and meets the requirements of green preservation technology [22,23]. In comparison to chitosan, Kadozan features a simpler preparation, wider application, and higher efficiency in practical applications of fruit preservation [23]. A previous study discovered that Kadozan treatment effectively inhibits disease caused by *Peronophythora litchii* infection and maintains the postharvest quality of litchi fruit [22]. Moreover, Kadozan treatment has also been employed to retard the development of pulp breakdown and pericarp browning in postharvest longan fruit [23,24].

However, very little is known regarding the effectiveness of Kadozan in preserving Indian jujube fruit after harvest. In this study, a series of the appearance quality and storability indicators were measured. These encompassed the decay index and weight loss, firmness, commercially acceptable fruit rate, respiration rate, relative electrolyte leakage rate, as well as appearance color change. Meanwhile, the nutritional components in Indian jujube fruit such as total soluble solids (TSS), TA, Vc, and total sugar contents were assessed. The objective of this study is to clarify the potential functions of Kadozan on postharvest quality and shelf-life of Indian jujube fruit.

## 2. Materials and Methods

### 2.1. Materials and Treatments

The commercially mature (~190 days after full bloom, with a fruit weight of 100 ± 5 g, firmness of 4.5 ± 0.5 N and total soluble solids of 12 ± 0.5%) Indian jujube fruit (‘Pingguozao’ *Ziziphus mauritiana* Lamk) was harvested from an orchard in Chang tai, Fujian province and delivered to the laboratory within one hour. The fruits were chosen without any signs of disease or mechanical damage, ensuring they were uniform in size and color. About 3200 Indian jujube fruits were selected for analysis in this study.

Kadozan, purchased from Lytone Enterprise, Inc., Shanghai, China, was used in this study. It had a deacetylation degree exceeding 95% and a molecular mass ranging from 20 to 30 kDa. Kadozan was diluted at ratio of 1:300, 1:600, 1:900 (V_Kadozan_:V_Kadozan+water_) with water and then thoroughly stirred. The Indian jujube fruits were arbitrarily split into four groups, with each group consisting of 800 fruits. The three groups of Indian jujube fruits were soaked in 1:300, 1:600 and 1:900 Kadozan solution for 5 min respectively, while the fourth group was submerged in distilled water for 5 min to serve as the control. Subsequently, to remove surface moisture, the fruit was air-dried for approximately 2 h. Afterward, the fruit was packed in polyethylene bags (thickness = 0.015 mm, 10 fruits/bag) and kept in a storage room for 18 days at 15 ± 1 °C with a relative humidity of 85%. Along with the storage, 6 bags of Indian jujube fruits were randomly selected every 3 days for liquid nitrogen quick-freezing and preservation for quality index measurement.

### 2.2. Determination of the Commercially Acceptable Rate, Decay Index, Firmness, and Weight Loss

Twenty Indian jujube fruits were subjected to estimate the commercially acceptable rate and weight loss, following the method outlined by Lin et al. [24]. In addition, the method of Chen et al. [3] was utilized to measure the decay index of twenty Indian jujube fruits. The results of the decay index, commercially acceptable rate and weight loss were expressed as percentage (%). The firmness of ten Indian jujube fruits was determined using a texture analyzer (Model CT3, Brookfield Engineering Laboratories Inc., Middleboro MA, USA) equipped with a TA 39 cylindrical flat bottom probe [25]. The measurement condition was puncture mode, with specific parameter settings as follows: trigger force 0.07 N, puncture speed 2 mm s^−1^, puncture depth 10 mm. The firmness was expressed as Newton (N).

### 2.3. Determination of Respiration Rate and Relative Electrolyte Leakage Rate

The respiration rate can be measured by the CO_2_ released per kilogram of fruit during respiration per hour according to Chen et al. [3]. The respiration rate of ten Indian jujube fruits was measured using a portable infrared CO_2_ gas analyzer (Model GXH-3051, Institute of Junfang Scientific Instrument, Beijing, China) and an infrared gas analyzer (IRGA). The result was represented as mg CO_2_ kg^−1^ h^−1^.

Two grams of pulp tissue discs (5 mm in diameter and 10 mm in thickness) from ten Indian jujube fruits were taken to evaluate the relative electrolyte leakage rate using a conductivity meter (Model DDS-307A, Shanghai Precision Scientific Instrument Co., Ltd., Shanghai, China), following the method of Xue et al. [26]. The relative electrolyte leakage rate was expressed as percentage (%).

### 2.4. Determination of the Peel Color, Chlorophyll, and Carotenoid Contents

The peel color from ten Indian jujube fruits was measured as previously mentioned by Hu et al. [27] with a chroma meter (Model WSC-S, Shanghai Precision Scientific Instrument Co., Ltd., Shanghai, China) on the opposite sides of the equatorial area of the fruit, using the CIE Lab scale (L*, a*, and b*). The equation, *h*° = ${tan}^{-1}\frac{b^{*}}{a^{*}}$, was conducted to compute the hue angle (*h*°).

The peel (2 g) from ten Indian jujube fruits was taken to assess the carotenoid and chlorophyll contents using the technique recommended by Lin et al. [24]. The peel tissue was crushed and extracted with 80% acetone for 3~5 min. Then, the extracted solution was filtered and used to determine the chlorophyll and carotenoid contents. The carotenoid and chlorophyll contents were defined as mg kg^−1^.

### 2.5. Determination the Contents of Total Sugar, TA, Vc, TSS

The pulp (2 g) from ten Indian jujube fruits was used to measure TSS and TA contents. Following the method of Raja and Shanmugasundaram. [28], TSS content was assessed with a digital refractometer (Model LC-DR-53B, Lichen Instrument Technology Co., Ltd., Shanghai, China). The value of TSS was depicted using %. The quantification of TA content was conducted by the titration of juice against NaOH and given as percent citric acid [24].

Ten Indian jujube fruits were used to measure vitamin C employing the methodology established by Shehata et al. [29]. The pulp tissue (1 g) of Indian jujube fruit was crushed and extracted with the oxalic acid solution. The vitamin C content was measured by titration with 2,6-dichlorophenol indophenol and expressed as mg kg^−1^. Total sugar content measurement was performed with 2 g pulp tissue from ten Indian jujube fruits using a phenol-sulfuric acid method according to Shehata et al. [29]. The total sugar level was defined as mg kg^−1^.

### 2.6. Statistical Analysis

All experimental measurements were performed in three replicates. The data were analyzed utilizing the IBM SPSS version 25.0, and the values were presented as the mean ± standard error in the figures. Origin 2021 and IBM SPSS were performed for correlation analysis and principal components analysis, respectively.

## 3. Results and Discussion

### 3.1. Effects of Kadozan Treatment on Fruit Firmness, Weight Loss, Commercially Acceptable Rate, and Decay Index in Indian Jujube Fruit

**Figure 1:**
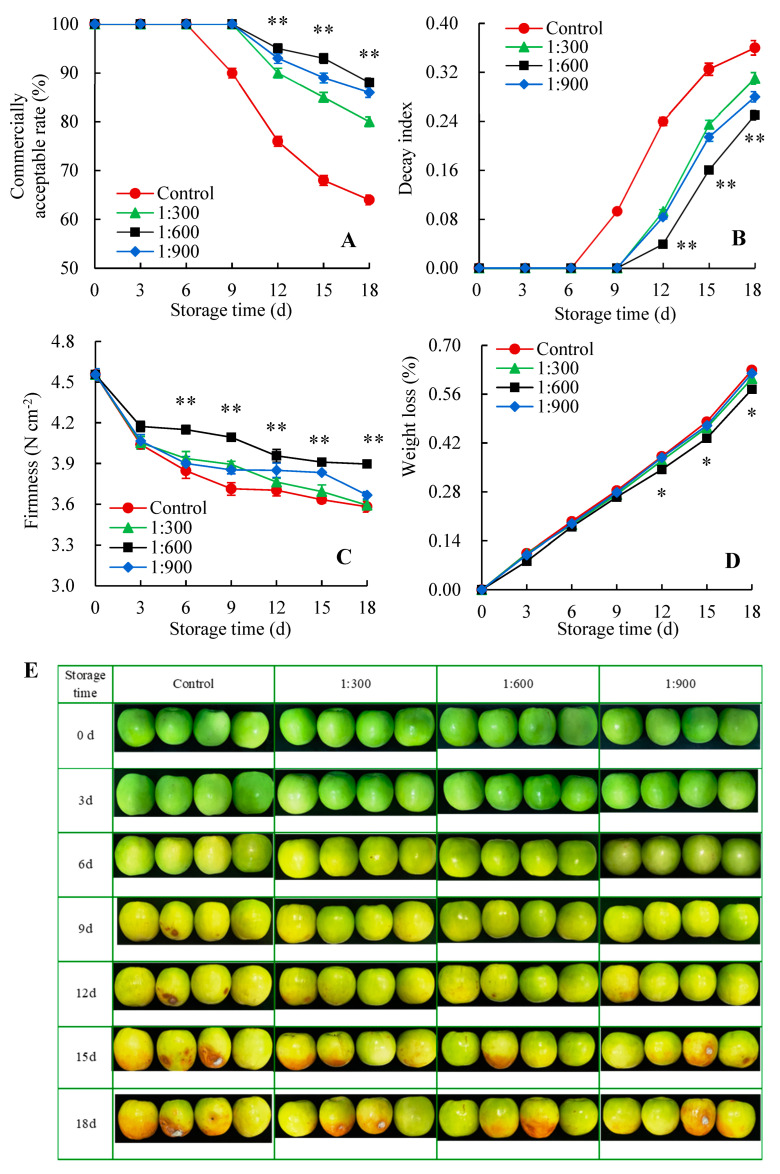
Changes in commercially acceptable rate (**A**), decay index (**B**), firmness (**C**), weight loss (**D**), and visual appearance (**E**) of Indian jujube fruit stored for 18 d at 15 ± 1 °C. The data were expressed as the mean ± standard error (*n* = 3). The significant differences between the 1:600 Kadozan-treated group and the control group within the same time were denoted by *p* values of <0.01 (**) and <0.05 (*). Red circular (●), control; green triangle (▲), 1:300 Kadozan treatment; black square (■), 1:600 Kadozan treatment; blue diamond (◆), 1:900 Kadozan treatment.

The commercially acceptable rate, weight loss, decay index, and firmness are the crucial quality indicators that reflect the commercial acceptance or economic value of fruit [30,31,32,33]. In this study, there was no change in the commercial acceptability rate of control fruit over the first 6 days of storage, afterward, dropping as a time of fruit storage progressed (Figure 1A). Nevertheless, the commercially acceptable rate in Kadozan-treated fruit began to decline on day 9, three days later than the control fruit. Moreover, Kadozan-treated group consistently exhibited a higher commercially acceptable rate in comparison with the control group throughout storage. The most effective treatment for maintaining a commercially acceptable rate was 1:600 Kadozan treatment, with 25.0%, 36.8%, and 37.5% higher than the control group at 12 d, 15 d, and 18 d, respectively.

As shown in Figure 1B, the decay index displayed a continuous and gradual increase in the treatment and control groups during storage. Nevertheless, after 6 days of storage, Kadozan-treated fruit showed a lower decay index than the control fruit. In addition, the decay index of fruit treated with Kadozan at 1:300, 1:600, and 1:900 was 13.89%, 30.56%, and 22.22% lower, respectively, compared to the control fruit on day 18 of storage. Compared to concentrations of 1:300 and 1:900, 1:600 Kadozan treatment exhibited superior effectiveness in inhibiting the decay of Indian jujube fruit.

As the storage period progressed, the control fruit showed a consistent decrease in firmness (Figure 1C). A similar declining trend was founded in Kadozan-treated fruit, but the Kadozan treatment retarded the decline of fruit firmness. Notably, 1:600 Kadozan treatment maintained higher fruit firmness during storage, which was 10.23% higher than that of the control on day 9 of storage.

The weight loss of treated and control Indian jujube fruit showed an increasing trend during storage (Figure 1D). However, the increase in the rate of weight loss was slowed by the Kadozan treatment. A similar result was observed in longan fruit, where Kadozan treatment effectively reduced weight loss [24]. This effect can be attributed to Kadozan’s ability to form a rapid and efficient barrier against gas exchange and water vapor loss, thereby enhancing water retention and minimizing postharvest weight loss [18].

Based on Figure 1E, it was found that the peel color in control Indian jujube fruit changed from green to yellow-green or even yellow as storage progressed. Specifically, after 9 days of storage, the control fruit exhibited a yellow-green color, with a small brown spot appearing on the peel. As time progressed, the quality deterioration of Indian jujube fruit became more severe. This was manifested by the enlargement of brown spot, softening of pulp tissue, and heightened susceptibility to decay-causing organisms. At the end of storage, majority fruits had developed yellow peel, large watery brown depressions, mold, soft texture, and exudates. These changes ultimately culminated in a pronounced moldy and sour smell of the fruit. However, Kadozan-treated fruit displayed a better appearance than the control fruit. The color of Kadozan-treated fruit turned yellow-green, and a slightly watery depression brown spot appeared on the peel on 12 days of storage, 3 days later than the control. According to the study conducted, Kadozan treatment could significantly suppress peel discoloration and fruit decay, and maintain a better appearance quality of Indian jujube fruit within storage time. Further comparison found that the appearance quality exhibited by the fruit treated with 1:600 Kadozan treatment was best throughout the storage period.

The above findings revealed that Kadozan treatment inhibited the decrease in firmness and weight loss, as well as the increase in decay index, suggesting it has an advantage in maintaining the commercially acceptable rate of Indian jujube fruit. These findings were consistent with Li et al. [34], treating with chitosan effectively inhibited the decay caused by *Colletotrichum gloeosporioides* and slowed down the decrease of firmness, resulting in an improvement in the storability of mango fruit. Another similar result was discovered by Zhong et al. [35], who reported that the Indian jujube fruit treated with chitosan reduced weight loss and retained fruit firmness during storage, thereby extending the shelf life of the fruit. Nevertheless, Kadozan has a significant preservation effect on Indian jujube fruit than chitosan. Kadozan-treated fruit exhibited higher firmness and lower weight loss compared to chitosan treatment. In addition, the storage life of fruit treated with chitosan was about 13–14 days, while the commercially acceptable rate of 1:600 Kadozan treatment reached 88% after 18 days of storage.

### 3.2. Effects of Kadozan Treatment on Relative Electrolyte Leakage Rate and Respiration Rate in Indian Jujube Fruit

**Figure 2:**
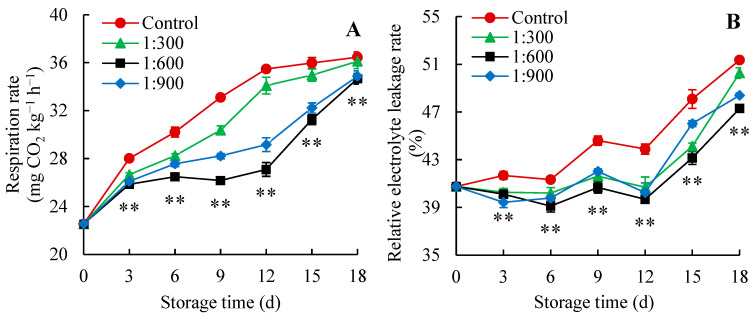
Changes in respiration rate (**A**) and relative electrolyte leakage rate (**B**) of Indian jujube stored for 18 d at 15 ± 1 °C. The data were expressed as the mean ± standard error (*n* = 3). The significant differences between the 1:600 Kadozan-treated group and the control group within the same time were denoted by *p* values of <0.01 (**). Red circular (●), control; green triangle (▲), 1:300 Kadozan treatment; black square (■), 1:600 Kadozan treatment; blue diamond (◆), 1:900 Kadozan treatment.

Respiration stands as the foremost physiological process in postharvest fruit during storage and serves as a crucial indicator of its physiological condition [36]. Previous research found that the fruit still undergoes respiration after picking, which consumes nutrients, accelerates fruit ripening and aging, and even leads to fruit decay [37,38,39]. Therefore, inhibiting the fruit respiration rate may reduce postharvest loss and extend shelf life. In this work, both the control and 1:300 Kadozan-treated fruits exhibited a rapid increase in respiration rate within 0–12 days, followed by a steady increase after 12 days of storage (Figure 2A). On the contrary, the respiration rate in the Indian jujube fruit treated with 1:600 and 1:900 Kadozan increased slowly over the first 12 days of storage and then increased rapidly after 12 days of storage. Further analysis found that Kadozan treatment could slow down the escalation in respiration rate, especially the respiration rate in the Indian jujube fruit treated with 1:600 Kadozan was consistently lower (*p* < 0.01) than that in the control fruit during the storage period. These findings showed that Kadozan treatment could inhibit the respiration rate, thus reducing nutrient consumption and maintaining the postharvest quality during Indian jujube fruit storage. Similar results were obtained by Chang et al. [40], who reported that the respiration rate in fresh-cut pear was effectively inhibited by chitosan treatment. Moreover, Li et al. [34] revealed that postharvest chitosan treatment reduced nutrition loss by decreasing the respiration rate, which subsequently improved the quality of mango fruit.

The relative electrolyte leakage rate was used as an index to evaluate the cell membrane integrity of the fruit. Once the cell membrane is damaged, its permeability increases, leading to a higher relative electrolyte leakage rate [30]. In the present study, with progressed storage duration, the relative electrolyte leakage rate showed an overall upward trend in the control and Kadozan-treated groups (Figure 2B). Nevertheless, Indian jujube fruit in the Kadozan-treated group consistently maintained a lower relative electrolyte leakage rate than those in the control group. Especially, it was found that the 1:600 Kadozan treatment exhibited superior effectiveness compared to the treatment of 1:300 Kadozan and 1:900 Kadozan in retarding the increase of relative electrolyte leakage rate during storage. Correlative analysis unveiled that the relative electrolyte leakage rate (Figure 2B) had a notable positive relationship (*p* < 0.01) with the decay index (Figure 1B) and weight loss (Figure 1D). This result suggested that the Kadozan treatment delayed the continuous increase of relative electrolyte leakage rate and maintained the membrane integrity in Indian jujube fruit, which helped to reduce the weight loss and decay index of the fruit. Similarly, Naveed et al. [41] suggested that the xanthan gum coating treatment could inhibit the increase in the relative electrolyte leakage rate of Indian jujube fruit, which may be related to reducing weight loss and disease incidence. Furthermore, Lin et al. [24] found that Kadozan treatment had an inhibitory effect on the relative electrolyte leakage rate, which possibly contributed to preserving the integrity of cell membrane and reduced the quality deterioration of longan fruit.

### 3.3. Effects of Kadozan Treatment on Peel Color, Carotenoid, and Chlorophyll Contents in Indian Jujube Fruit

**Figure 3:**
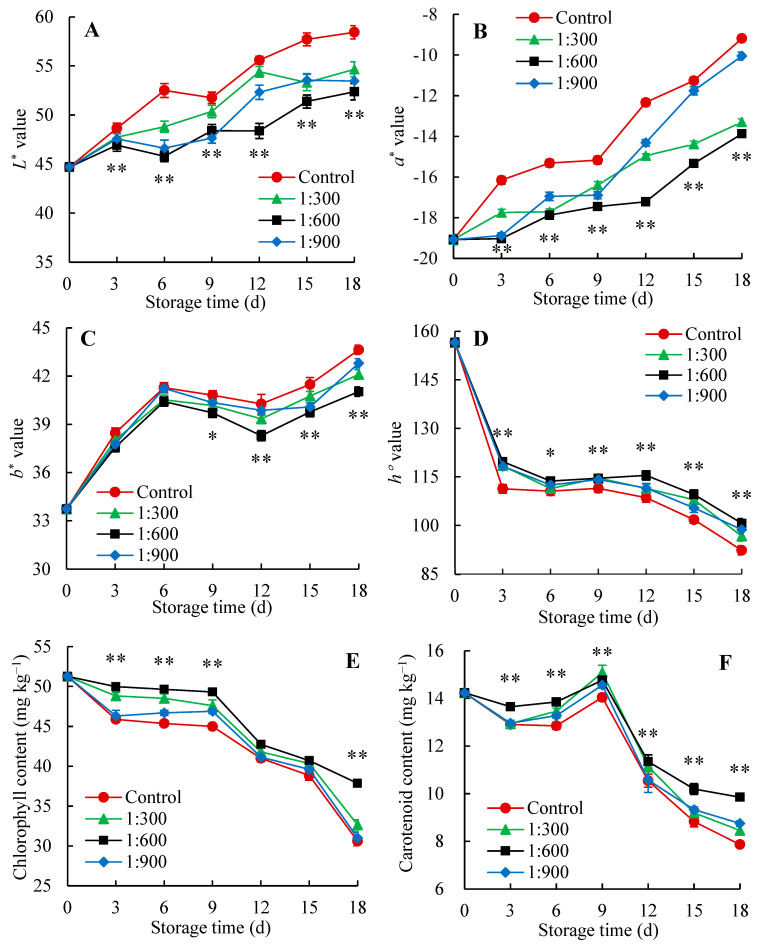
Changes in L* (**A**), a* (**B**), b* (**C**), *h*° (**D**), chlorophyll (**E**), and carotenoid (**F**) of Indian jujube fruit stored for 18 d at 15 ± 1 °C. The data were expressed as the mean ± standard error (*n* = 3). The significant differences between the 1:600 Kadozan-treated group and the control group within the same time were denoted by *p* values of <0.01 (**) and <0.05 (*). Red circular (●), control; green triangle (▲), 1:300 Kadozan treatment; black square (■), 1:600 Kadozan treatment; blue diamond (◆), 1:900 Kadozan treatment.

Fruit color is a critical quality factor that determines its market appeal and consumer preference [41]. In our work, the change in peel color of Indian jujube fruit was assessed by measuring the values of a*, b*, L*, and *h*°. The a*, b*, and L* values are graded from green (−a*) to red (+a*), blue (−b*) to yellow (+b*), and black (0) to white (100), respectively. As illustrated in Figure 3A,B, the L* and a* values showed a progressive increase in the fruit with or without Kadozan treatment as the storage duration extended. For b* value, a significant increase was shown in the first 6 days, rising from 33.73 to 41.28. Subsequently, there was a slight decline from day 6 to day 12, followed by a rapid upsurge within the 12–18 days of storage (Figure 3C).

In contrast, *h*° value exhibited a sharp decline during the initial storage period (0–3 days), remained stable with minor fluctuations from day 3 to day 9, and subsequently experienced a rapid drop after storage day 9 (Figure 3D). Further analysis indicated that the Kadozan treatment exhibited superior effectiveness in delaying the decrease in *h*° value and the increase in L*, b* and a* values than those in the control fruit, especially the 1:600 Kadozan treatment. The aforementioned results demonstrated that the Kadozan treatment has a crucial effect on preserving the superior appearance of Indian jujube fruit across storage.

Carotenoid and chlorophyll serve as the major pigments accountable for the coloration of Indian jujube fruit [41]. As shown in Figure 3E, the chlorophyll content showed a decrease throughout the storage duration in Kadozan-treated and control groups (Figure 3E). However, Indian jujube fruit in the Kadozan-treated group consistently maintained higher chlorophyll content than those in the control group. In this study, the application of 1:600 Kadozan treatment was found to significantly retard the decline in chlorophyll, especially at the end of storage which was 23.7% higher than that of the control group. Figure 3F shows that the level of carotenoid in the Indian jujube fruit without Kadozan treatment declined slowly within the earlier storage duration (0–3 d), followed by a rise from day 3 to day 9, and finally slipped quickly after day 9. A similar pattern of the carotenoid level was observed in the Kadozan-treated group. Further comparison revealed the carotenoid content in the 1:600 Kadozan-treated group was considerably higher (*p* < 0.01) than in the control group over the whole storage period.

The above findings showed that the Kadozan treatment preserved the appearance quality of Indian jujube fruit by suppressing the change of peel color during storage. These effects could be ascribed to Kadozan treatment resulting in lower a*, b*, and L* values and a higher *h*° value, as well as maintained higher chlorophyll and carotenoid contents. Similar to this work, the decrease in chlorophyll and carotenoid contents in longan fruit treated with Kadozan was also significantly inhibited [24]. Jiang et al. [42] found that Kadozan treatment suppressed a decrease in a*, b*, and L* values and maintained the appearance color of litchi fruit. For postharvest Indian jujube fruit, hexanal treatment exhibited higher a*, b*, and L* values as compared with control values which contributed to better maintaining the peel color attributes [43]. In addition, the grape coated with chitosan showed higher L* and *h*° values, eventually delaying the change of fruit color as compared with control [44].

### 3.4. Effects of Kadozan Treatment on TA, TSS, Vc and Total Sugar Contents in Indian Jujube Fruit

**Figure 4:**
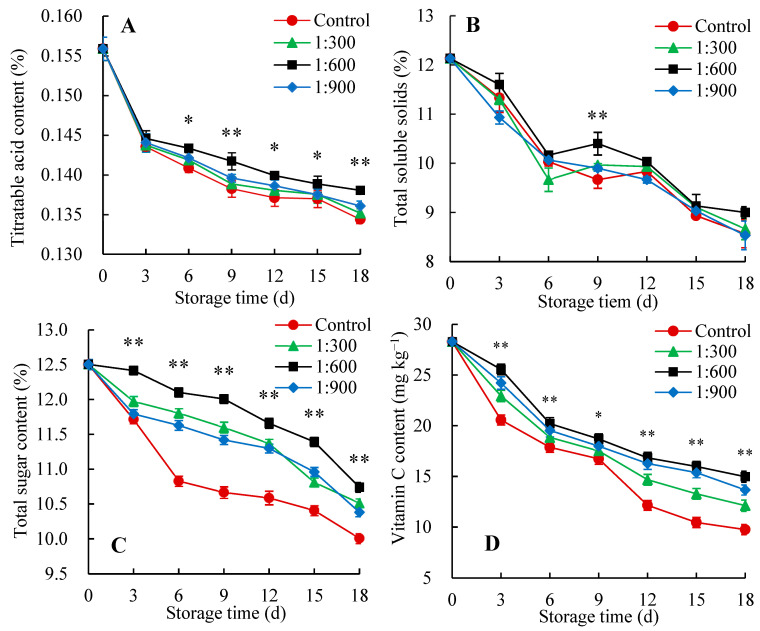
Changes in TA (**A**), TSS (**B**), total sugar content (**C**), and Vc (**D**) of Indian jujube fruit stored for 18 d at 15 ± 1 °C. The data were expressed as the mean ± standard error (*n* = 3). The significant differences between the 1:600 Kadozan-treated group and the control group within the same time were denoted by *p* values of <0.01 (**) and <0.05 (*). Red circular (●), control; green triangle (▲), 1:300 Kadozan treatment; black square (■), 1:600 Kadozan treatment; blue diamond (◆), 1:900 Kadozan treatment.

TA, Vc, TSS and total sugar contents are important quality indicators that influence the flavor and nutritional value of the fruit [16]. As depicted in Figure 4A, the TA content in the Kadozan-treated and control groups gradually decreased with progressed storage. Compared to the control fruit, Indian jujube fruit treated by Kadozan maintained higher TA content during storage. Especially, after 6 days of storage, the decreased trend of TA content was more remarkable in the control fruit in comparison to 1:600 Kadozan-treated fruit. The TSS content exhibited a sharp decrease during the initial 6 days of storage, then showed a slight increase at 6–9 days, and finally rapidly decreased (Figure 4B). The decrease was less pronounced in Kadozan-treated fruit compared to the control fruit. Nevertheless, there was no significant difference in TSS content during storage regardless of treatments except on days 9 (*p* < 0.05). For total sugar and Vc content, both Kadozan-treated and control groups exhibited a consistent decrease (Figure 4C,D). However, the Indian jujube fruit in the Kadozan-treated group consistently maintained higher total sugar and Vc content than those in the control group. Further comparison revealed that 1:600 Kadozan treatment recorded the greatest effects on maintaining the total sugar and Vc content in Indian jujube fruit.

Correlation analysis demonstrated that the TA, Vc, TSS, and total sugar contents in Kadozan-treated and control fruit were negatively correlated (*p* < 0.01) with the respiration rate. Therefore, it can be inferred that Kadozan treatment suppressed the respiration rate of Indian jujube fruit which restricts the reduction of TA, TSS, total sugar, and Vc contents eventually resulting in markedly higher fruit quality than the control. Similar to this work, the mango coated with chitosan showed higher TSS and soluble sugar contents as compared with control fruit, which possibly helped to improve the postharvest quality of the fruit [45]. In another work, Lin et al. [24] reported the higher TSS, Vc, and total sugar contents in Kadozan-treated fruit could be attributed to delayed respiration rate, and thereby maintaining the higher quality of longan fruit.

### 3.5. Principal Component Analysis and Comprehensive Evaluation

**Figure 5:**
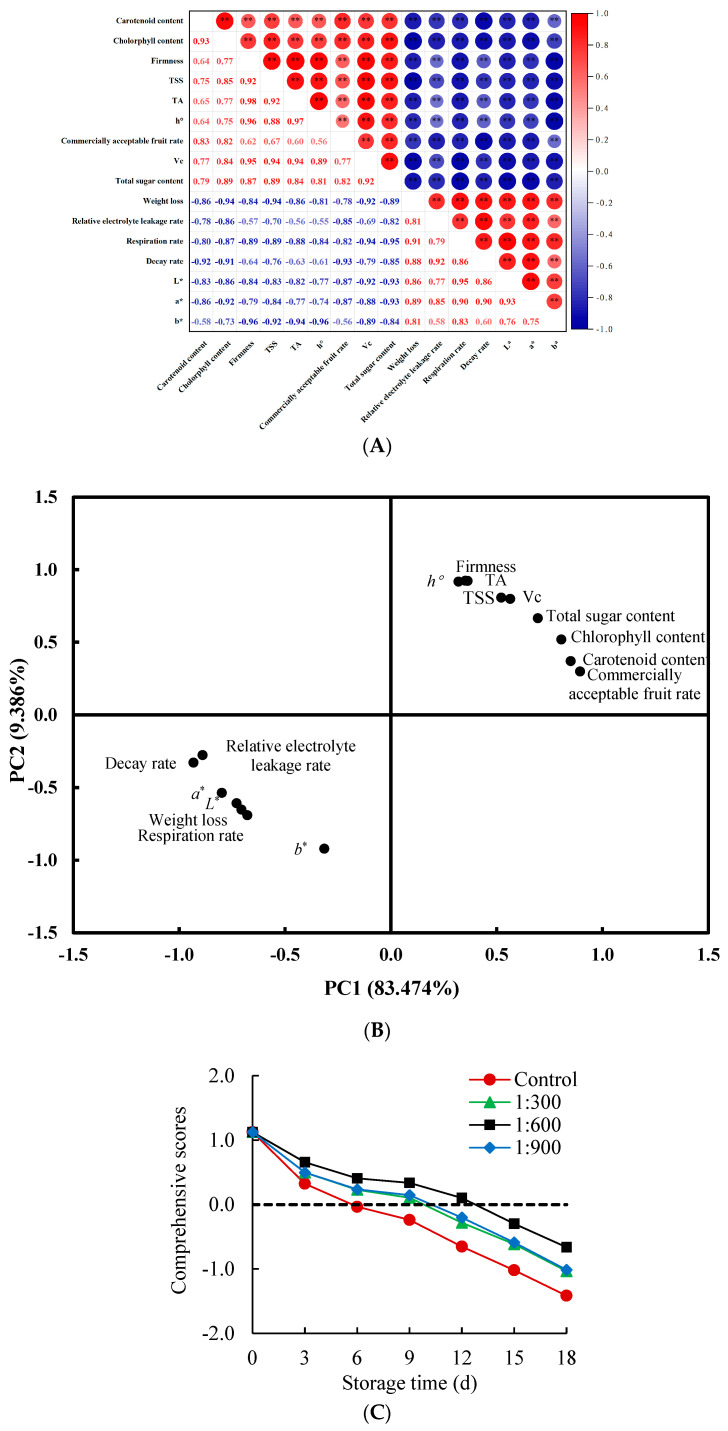
Correlation matrix (**A**), loadings plot (**B**) and comprehensive scores (**C**). The significant differences between the 1:600 Kadozan-treated group and the control group within the same time were denoted by *p* values of <0.01 (**). Red circular (●), control; green triangle (▲), 1:300 Kadozan treatment; black square (■), 1:600 Kadozan treatment; blue diamond (◆), 1:900 Kadozan treatment.

Exploring the possible mechanism of the efficacy of Kadozan treatment on the storage quality in Indian jujube fruit through principal component analysis. The correlation matrix (Figure 5A) indicated that the commercially acceptable fruit rate had a notable positive relationship (*p* < 0.01) with carotenoid content, chlorophyll content, firmness, TSS, TA, *h*°, Vc, and total sugar content, while having a negative relationship (*p* < 0.01) with decay index, respiration rate, relative electrolyte leakage rate, weight loss, L*, a*, and b* (Supplementary Table S1). These results indicated that the quality maintenance of Indian jujube fruit was not only related to the reduction of decay index, respiration rate, relative electrolyte leakage rate, weight loss, L*, a*, and b*, but also to the maintenance of carotenoid content, chlorophyll content, firmness, TSS, TA, *h°*, Vc, and total sugar content.

The related indicators of storability and quality of Indian jujube fruit during storage were standardized, and then principal component analysis was performed. A significant difference in variance (*p* < 0.01) was determined based on Bartlett’s sphericity test, with a Kaiser–Meyer–Olkin measurement of 0.835 (over 0.8= excellent), indicating that the data meet the requirements of principal component analysis (Supplementary Table S2). The eigenvalues of PC1 (13.356) and PC2 (1.502) were both greater than 1, and the cumulative contribution rate of PC1 (83.474%) and PC2 (9.386%) was 92.860%, which could be used to illustrate the quality and physiology changes of Indian jujube fruit through the whole storage (Supplementary Table S3). Therefore, two-dimensional scatter plots were created based on the scores of each quality index on PC1 and PC2. As depicted in Figure 5B, the *h°* value, firmness, TA, TSS, Vc, total sugar content, chlorophyll content, carotenoid content, and commercially acceptable fruit rate all fell within the [0, 1.5] interval of PC1 and PC2, which were positively correlated with PC1 and PC2. However, the relative electrolyte leakage rate, decay rate, a* value, b* value, L* value, weight loss, and respiration rate all fell within the [0, −1.5] interval of PC1 and PC2, suggesting that these seven indicators exhibited a negative correlation with PC1 and PC2. The larger PC1 and PC2, the higher *h*° value, firmness, TA, TSS, Vc, total sugar content, chlorophyll content, carotenoid content, and commercially acceptable fruit rate, the lower relative electrolyte leakage rate, decay rate, weight loss, and respiration rate, indicating the better quality and storability of fruit. Therefore, PC1 and PC2 can be defined as the quality and storability indicators of Indian jujube fruit.

For a better depiction of the alterations in the quality of Indian jujube fruit treated with Kadozan throughout storage, linear equations were developed and used to calculate composite scores (Supplementary Table S4). Carotenoid content (X_1_), chlorophyll content (X_2_), firmness (X_3_), relative electrolyte leakage rate (X_4_), respiration rate (X_5_), TSS content (X_6_), TA content (X_7_), decay index (X_8_), L* value (X_9_), a* value (X_10_), b* value (X_11_), *h*° (X_12_), commercially acceptable fruit rate (X_13_), weight loss (X_14_), Vc content (X_15_), and total sugar content (X_16_) were used as the data source for analysis. The equations used to calculate these scores are as follows:

Y_1_ = 0.203X_1_ + 0.142X_2_ − 0.140X_3_ − 0.245X_4_ − 0.046X_5_ − 0.047X_6_ − 0.144X_7_ − 0.246X_8_ − 0.088X_9_ − 0.135X_10_ + 0.156X_11_ − 0.154X_12_ + 0.240X_13_ − 0.067X_14_ − 0.029X_15_ + 0.058X_16_ (1)

Y_2_ = −0.113X_1_ − 0.044X_2_ + 0.238X_3_ + 0.160X_4_ − 0.057X_5_ + 0.148X_6_ + 0.241X_7_ + 0.154X_8_ − 0.011X_9_ + 0.036X_10_ − 0.251X_11_ + 0.249X_12_ − 0.152X_13_ − 0.034X_14_ + 0.132X_15_ + 0.043X_16_ (2)

The equation for calculating the comprehensive score was Y = 0.5039Y_1_ + 0.4961Y_2_, where Y represented the comprehensive score, Y1 and Y2 corresponded to the PC1 and PC2, and Xi (X_1_–X_14_) represented the standardized data. As shown in Figure 5C, the comprehensive score of Kadozan and control groups remarkably decreased along with the storage time extended. A further comparison found that the comprehensive score in the Kadozan-treated group was higher than in the control group, especially in 1:600 Kadozan-treated group. These results established that Kadozan treatment remarkably improved the quality and storability of Indian jujube fruit. The effect of different treatments in the order: 1:600 Kadozan treatment > 1:900 Kadozan treatment > 1:300 Kadozan treatment > control group.

## 4. Conclusions

**Figure 6:**
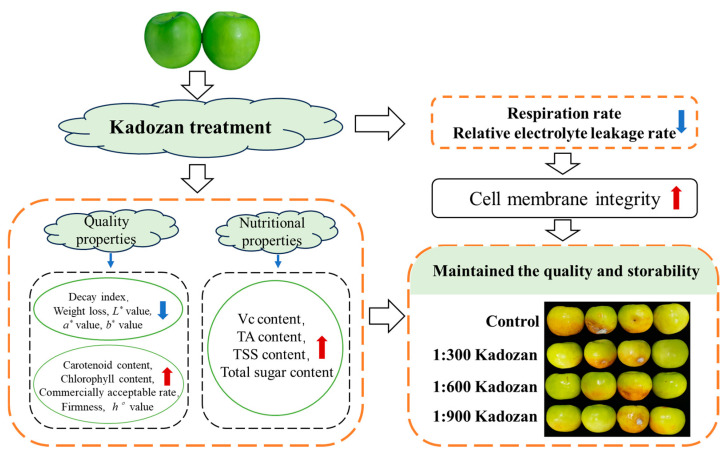
The schematic diagram of Kadozan treatment to improve the postharvest quality of Indian jujube fruit during storage. ↑ indicated the higher levels of the physicochemical indicators in the Kadozan-treated fruit than the control fruit; ↓ indicated the lower levels of the physicochemical indicators in the Kadozan-treated fruit than the control fruit.

In this study, Kadozan treatment was found to effectively improve the postharvest quality and shelf life of Indian jujube fruit. On the one hand, Kadozan treatment was effective in the delay of color changes and the maintenance of superior appearance quality. Secondly, Kadozan treatment was effective in reducing nutrient loss by inhibiting respiration of Indian jujube fruit. In addition, Kadozan treatment maintained cell membrane integrity by suppressing fruit respiration and mitigating cell membrane damage. This is conducive to ensuring the physiological and metabolic processes of the fruit and then improving the storage longevity of Indian jujube fruit. The correlation matrix, loadings plot, and comprehensive scores further confirmed that the application of Kadozan treatment significantly improved the quality and storability of Indian jujube fruit compared to the control, and the schematic diagram was generalized in Figure 6. In particular, the 1:600 Kadozan treatment showed the greatest efficacy compared to the 1:300 and 1:900 concentrations. Considering these results, we advocate the use of 1:600 Kadozan as an alternative method for preserving postharvest quality and extending the shelf life of Indian jujube fruit.

## Data Availability

The original contributions presented in the study are included in the article, further inquiries can be directed to the corresponding author.

## Supplementary Materials

The following supporting information can be downloaded at: https://www.mdpi.com/article/10.3390/foods14020266/s1, Table S1: Correlation Matrix; Table S2: KMO and Bartlett’s test; Table S3: Total variance explained; Table S4: Component score coefficient matrix.
