# Supplementary material for: Kadozan Chitosan Formulation Enhances Postharvest Quality of Fresh Indian Jujube Fruit
Source: Foods. 2025 Jan 15;14(2):266. doi: 10.3390/foods14020266 (PMC11765311; doi:10.3390/foods14020266)
Supplement: Supplementary file 1 [file foods-14-00266-s001.zip › foods-3386189-supplementary.pdf]

Supplementary Table S1. Correlation Matrix.

	Carotenoid content	Cholorphyll content	Firmness	Relative electrolyte leakage rate	Respiration rate	TSS	TA	Decay index	L^*	a^*	b^*	$h°$	Commercially acceptable fruit rate	Weight loss	Vc	Total sugar content
**Carotenoid content**	1.000	0.928	0.640	−0.778	−0.803	0.753	0.654	−0.921	−0.826	−0.857	−0.583	0.645	0.831	−0.863	0.766	0.790
**Cholorphyll content**	0.928	1.000	0.766	−0.858	−0.874	0.849	0.767	−0.910	−0.861	−0.918	−0.730	0.749	0.818	−0.942	0.844	0.893
**Firmness**	0.640	0.766	1.000	−0.568	−0.891	0.921	0.985	−0.636	−0.841	−0.787	−0.959	0.956	0.621	−0.841	0.946	0.872
**Relative electrolyte leakage rate**	−0.778	−0.858	−0.568	1.000	0.789	−0.697	−0.556	0.924	0.770	0.853	0.580	−0.553	−0.848	0.811	−0.688	−0.820
**Respiration rate**	−0.803	−0.874	−0.891	0.789	1.000	−0.891	−0.878	0.863	0.949	0.899	0.830	−0.836	−0.822	0.907	−0.941	−0.949
**TSS**	0.753	0.849	0.921	−0.697	−0.891	1.000	0.916	−0.765	−0.828	−0.835	−0.915	0.885	0.674	−0.941	0.943	0.886
**TA**	0.654	0.767	0.985	−0.556	−0.878	0.916	1.000	−0.633	−0.825	−0.766	−0.937	0.972	0.596	−0.856	0.938	0.845
**Decay index**	−0.921	−0.910	−0.636	0.924	0.863	−0.765	−0.633	1.000	0.858	0.901	0.598	−0.606	−0.934	0.884	−0.786	−0.847
L^*	−0.826	−0.861	−0.841	0.770	0.949	−0.828	−0.825	0.858	1.000	0.926	0.758	−0.774	−0.875	0.864	−0.917	−0.929
a^*	−0.857	−0.918	−0.787	0.853	0.899	−0.835	−0.766	0.901	0.926	1.000	0.754	−0.736	−0.865	0.894	−0.877	−0.930
b^*	−0.583	−0.730	−0.959	0.580	0.830	−0.915	−0.937	0.598	0.758	0.754	1.000	−0.956	−0.562	0.807	−0.894	−0.841
$h°$	0.645	0.749	0.956	−0.553	−0.836	0.885	0.972	−0.606	−0.774	−0.736	−0.956	1.000	0.558	−0.813	0.891	0.811
**Commercially acceptable fruit rate**	0.831	0.818	0.621	−0.848	−0.822	0.674	0.596	−0.934	−0.875	−0.865	−0.562	0.558	1.000	−0.779	0.774	0.821
**Weight loss**	−0.863	−0.942	−0.841	0.811	0.907	−0.941	−0.856	0.884	0.864	0.894	0.807	−0.813	−0.779	1.000	−0.923	−0.886
**Vc**	0.766	0.844	0.946	−0.688	−0.941	0.943	0.938	−0.786	−0.917	−0.877	−0.894	0.891	0.774	−0.923	1.000	0.917
**Total sugar content**	0.790	0.893	0.872	−0.820	−0.949	0.886	0.845	−0.847	−0.929	−0.930	−0.841	0.811	0.821	−0.886	0.917	1.000

Supplementary Table S2. KMO and Bartlett's Test.

Kaiser-Meyer-Olkin Measure of Sampling Adequacy.		0.835
Bartlett's Test of Sphericity	Approx. Chi-Square	929.336
	df	120
	Sig.	0.000

Supplementary Table S3. Total Variance Explained.

Component	Initial Eigenvalues			Extraction Sums of Squared Loadings			Rotation Sums of Squared Loadings		
	Total	Variance contribution rate %	Cumulative variance contribution %	Total	Variance contribution rate %	Cumulative variance contribution %	Total	Variance contribution rate %	Cumulative variance contribution%
1	13.356	83.474	83.474	13.356	83.474	83.474	7.487	46.795	46.795
2	1.502	9.386	92.860	1.502	9.386	92.860	7.371	46.066	92.860
3	0.327	2.041	94.902						
4	0.257	1.605	96.507						
5	0.135	0.844	97.351						
6	0.124	0.774	98.125						
7	0.091	0.571	98.696						
8	0.062	0.387	99.083						
9	0.050	0.311	99.394						
10	0.030	0.190	99.584						
11	0.024	0.151	99.735						
12	0.018	0.115	99.850						
13	0.013	0.079	99.929						
14	0.007	0.044	99.972						
15	0.003	0.018	99.990						
16	0.002	0.010	100.000						

Supplementary Table S4. Component Score Coefficient Matrix.

	Component	
	1	2
Carotenoid content	0.203	−0.113
Cholorphyll content	0.142	−0.044
Firmness	−0.140	0.238
Relative electrolyte leakage rate	−0.245	0.160
Respiration rate	−0.046	−0.057
TSS	−0.047	0.148
TA	−0.144	0.241
Decay index	−0.246	0.154
L^*	−0.088	−0.011
a^*	−0.135	0.036
b^*	0.156	−0.251
$h°$	−0.154	0.249
Commercially acceptable fruit rate	0.240	−0.152
Weight loss	−0.067	−0.034
Vc	−0.029	0.132
Total sugar content	0.058	0.043
